# Using the serratus anterior free flap for dynamic facial reanimation: Systematic review

**DOI:** 10.1002/hed.27219

**Published:** 2022-10-19

**Authors:** Stefan Janik, Blazen Marijic, Muhammad Faisal, Stefan Grasl, Chieh‐Han J. Tzou, Andres Rodriquez‐Lorenzo, Rudolf Seemann, Matthias Leonhard, Boban M. Erovic

**Affiliations:** ^1^ Department of Otorhinolaryngology – Head and Neck Surgery Medical University of Vienna Vienna Austria; ^2^ Institute for Head and Neck Diseases Evangelical Hospital Vienna Vienna Austria; ^3^ Department of Otorhinolaryngology – Head and Neck Surgery University of Rijeka Rijeka Croatia; ^4^ Shaukat Khanum Memorial Cancer Hospital Lahore Pakistan; ^5^ Division of Plastic and Reconstructive Surgery, Department of Surgery Hospital of Divine Savior Vienna Austria; ^6^ Faculty of Medicine Sigmund Freud University Vienna Austria; ^7^ TZOU MEDICAL Vienna Austria; ^8^ Department of Plastic and Maxillofacial Surgery Uppsala University Hospital Uppsala Sweden; ^9^ Department of Surgical Sciences Uppsala University Hospital Uppsala Sweden

**Keywords:** ablative midface surgery, composite flap, dynamic facial reanimation, parotidectomy, serratus anterior free flap

## Abstract

It was the purpose of this study to evaluate the role of the serratus anterior free flap (SAFF) with its long thoracic nerve (LTN) as composite flap for dynamic facial reanimation. A total of 10 studies, published between 2004 and 2021, met inclusion criteria. Clinical data of 48 patients were used for the systematic review and analysis. One to three slips were used, mainly as one‐stage procedures (*n* = 39; 81.3%), to create different force vectors. Single or double innervated muscle transfers were utilized in 32 (66.7%) and 16 (33.3%) cases with additionally harvested skin paddles in 4 (8.3%) patients. The LTN was mostly anastomosed to the ipsilateral masseteric nerve (45.8%; *n* = 22) or to remaining facial nerve branches (37.5%; *n* = 18), while cross‐facial‐nerve‐grafting was rarely used (16.7%; *n* = 8). The SAFF as composite flap with different force vectors proved to be a good candidate for immediate dynamic facial reanimation after any midface defects.

**Abbreviations**
CFNG: cross facial nerve graft
FN: facial nerve
GMFT: gracilis muscle free transfer
LD: latissimus dorsi muscle
LTN: long thoracic nerve
MN: masseteric nerve
SA: serratus anterior muscle
SAFF: serratus anterior free flap
TDN: thoracodorsal nerve

## 1 INTRODUCTION

Ablative surgery of the parotid gland or midface tumors may end up in partial or complete facial palsies with complex soft tissue defects. Although oncological aspects must of course predominate, head and neck surgeons are also expected to achieve satisfactory functional and aesthetic results by offering advanced surgical facial nerve reanimation techniques.

Primary tension‐free coaptation of the facial nerve offers the best situation for recovery followed by cable grafting with donor nerves, which is, however, seldom possible in case of large tumor resections.^1^, ^2^ Static procedures, like brow lift, tarsorrhaphy, or gold weight insertion are especially sufficient for upper face reconstruction, while sufficient oral competence, speech, or smiling can only be achieved by dynamic facial repair.^3^, ^4^, ^5^

Hence, in terms of functionality, dynamic facial reanimation should be favored over static or non‐neurotized flap reconstructions. In long‐term facial palsies, the gracilis muscle free transfer (GMFT) is mostly utilized either as one‐stage procedure with connection to the ipsilateral masseteric nerve (MN) or as two‐stage procedure with cross‐facial nerve grafting (CFNG) followed by muscle transfer approximately 1 year after nerve grafting.^6^, ^7^ Despite great results, those long‐standing procedures are less suited to immediate facial reanimation after radical parotidectomy in patients with limited life expectancy and need for adjuvant therapy.^7^, ^8^

Consequently, free muscle transfers with sufficient motoric donor nerves and reliable skin islands have been of great interest for single‐stage dynamic facial and soft tissue repair after ablative midface/parotid resections. Up to now various free flaps have been described including the vastus lateralis, the latissimus dorsi (LD) or the serratus anterior free flap (SAFF).^7^, ^9^, ^10^, ^11^, ^12^ Particularly the SAFF has already found many applications in head and neck reconstruction since its first description four decades ago.^12^, ^13^, ^14^, ^15^, ^16^ The SAFF poses indeed a great candidate for dynamic facial reanimation due to (i) its long neurovascular pedicle consisting of the long thoracic nerve (LTN), (ii) the serratus branch of the thoracodorsal vessels, (iii) the option of skin paddles, and finally (iv) the harvest of up to five thin slips for multiple force vectors united in one pedicle.^17^, ^18^

Despite these obvious advantages of the composite SAFF for dynamic facial reanimation, only limited data have been published to date. Therefore, the aim of this study was to review and analyze current literature regarding applications of the SAFF for dynamic facial reanimation and to provide an overview of limitations and advantages of this versatile flap.

## 2 MATERIAL AND METHODS

### 2.1 Literature review

We have performed a comprehensive literature research in PubMed, Scopus, and Google scholar for papers published until June 1, 2021. Following keywords were applied “serratus anterior free flap,” “SAFF,” “serratus anterior” in combination with “facial reconstruction” and “facial reanimation.” In addition, we have reviewed references of appropriate and related articles.

### 2.2 Inclusion and exclusion criteria

Following criteria had to be fulfilled for inclusion in our analysis: (1) use of serratus anterior free flap; (2) dynamic facial reanimation; (3) free available studies published in English; (4) detailed clinical information regarding reconstruction. Consequently, studies reporting of (1) pedicled serratus anterior flaps; (2) absence of dynamic facial reconstruction; (3) letters, commentaries, editorials which did not contain valuable original data were excluded.

### 2.3 Search findings, data extraction, and outcome

**FIGURE 1:**
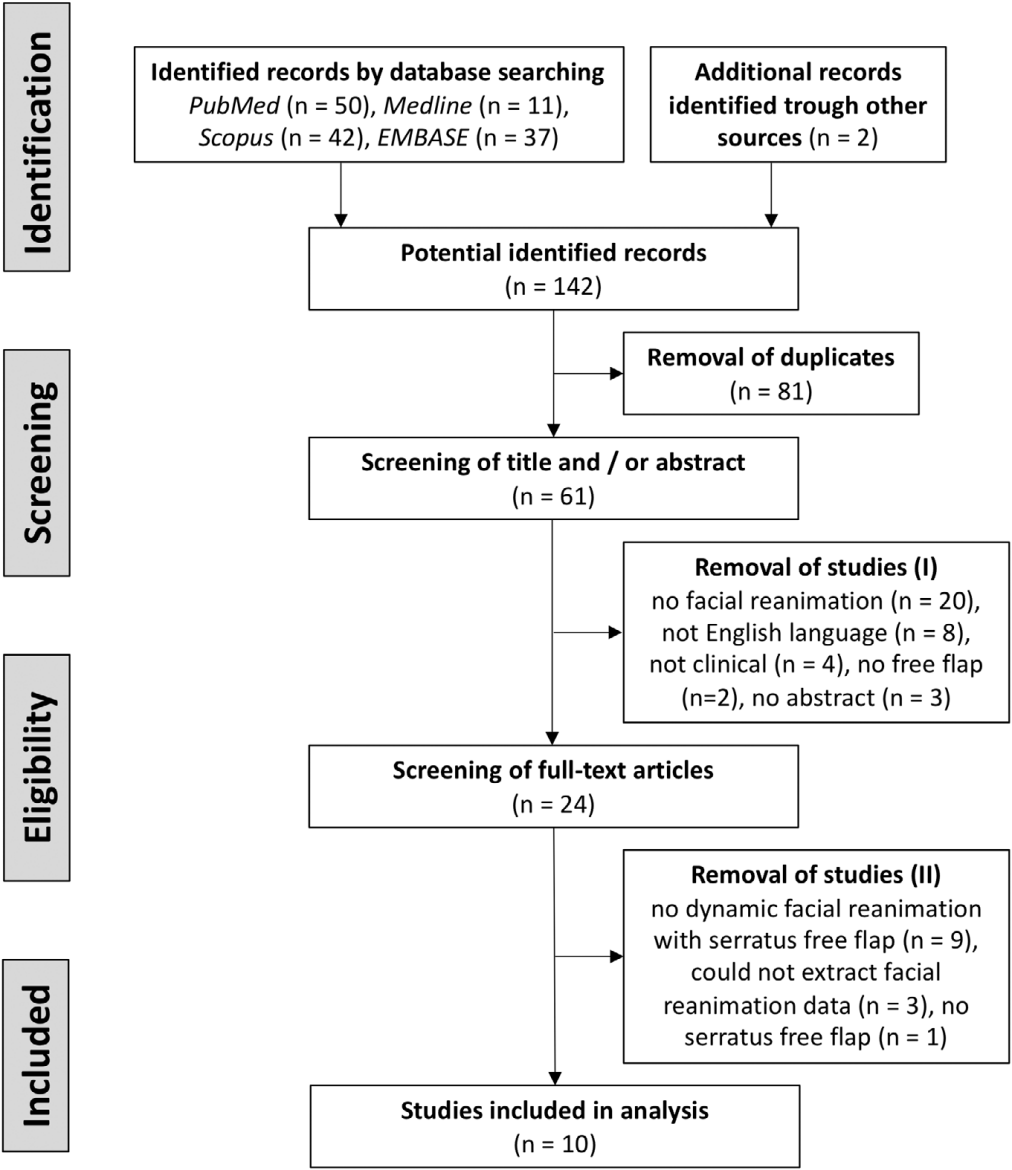
Flowchart

Titles and abstracts were screened independently by two authors (Stefan Janik and Blazen Marijic) first. All eligible articles and those with uncertain eligibility were retrieved for full‐text review. Disagreements were resolved by consensus or a third assessor (Boban M. Erovic). PRISMA guidelines were applied for identification of appropriate studies (Figure 1).^19^ Beside sociodemographic data (age, sex), we extracted data regarding reason and duration of facial palsy, if single or two‐stage reconstruction approach was performed, number of used muscle slips, use of skin‐paddle, selection of recipient nerves for nerval coaptation, use of cross‐facial nerve graft (CFNG), donor site morbidity, and outcome.

### 2.4 Statistical methods

SPSS (version 27; IBM Corp., Armonk, NY) was used for statistical analysis of data. Descriptive analyses were mainly applied and absolute numbers with corresponding percentages within brackets are indicated. Metric variables are indicated as mean ± standard deviation (SD) within the result section if not otherwise specified.

## 3 RESULTS

### 3.1 Study cohort

In total, 10 studies met eligibility criteria and were included in our systematic review (Figure 1). Our cohort consisted of 48 patients with a mean age of 38.8 years (range: 5–89 years) and a female predominance of 61.9%. Facial palsy mainly resulted from tumor resections (57.1%) followed by congenital (19.0%), infectious (14.3%), and traumatic causes (9.5%). Facial palsy persisted for a mean time of 4 years (range 0–10 years) (Table 1).

**TABLE 1:** Characteristics of included studies

Included study	Country	Nr.	F:M	Age	Etiology			
					Congenital	Oncologic	Trauma	Infectious
Sakuma et al.^25^	Japan	1	1:0	67	0 (0.0)	1 (100.0)	0 (0.0)	0 (0.0)
Watanabe et al.^26^	Japan	7	6:1	51	0 (0.0)	7 (100.0)	0 (0.0)	0 (0.0)
Sakuma et al.^24^	Japan	12	8:4	36	2 (16.7)	4 (33.3)	1 (8.3)	5 (41.7)
Matsumine et al.^27^	Japan	2	1:1	43	0 (0.0)	1 (50.0)	1 (50.0)	0 (0.0)
Gundeslioglu et al.^44^	Turkey	5	2:3	68	0 (0.0)	5 (100.0)	0 (0.0)	0 (0.0)
Cheng et al.^29^	China	4	1:3	19	4 (100.0)	0 (0.0)	0 (0.0)	0 (0.0)
Tang et al.^45^	China	6	4:2	25	2 (33.3)	2 (33.3)	1 (16.7)	1 (16.7)
Leonetti et al.^21^	USA	3	2:1	18	0 (0.0)	3 (100.0)	0 (0.0)	0 (0.0)
Yoleri^28^	Turkey	2	1:1	20	0 (0.0)	1 (50.0)	1 (50.0)	0 (0.0)
Ylä‐Kotola et al.^46^	Finland	6	NA	NA	NA	NA	NA	NA
Total		48	26:16	38.8	8 (19.0)	24 (57.1)	4 (9.5)	6 (14.3)

*Note*: Total number of included patients (Nr.), female to male ratio (F:M), mean patient age (in years), and etiology are indicated. Sufficient data were not available (NA) for one included study.

### 3.2 Timeline of publications

All studies were published between 2004 and 2021 and originated mostly from Asia (*n* = 8; 80%) followed equally by Europe (*n* = 1; 10%) and North America (*n* = 1; 10%). Considering timeline of publications, half of the studies were published between 2004 and 2010 (21 cases), and the remaining cases were published between 2017 and 2021 (27 cases). Of note, almost half of data (*n* = 22; 45.8%) were published within the past 4 years by Japanese groups.

### 3.3 Characteristics of used serratus anterior free flaps

Dynamic facial reanimation was accomplished with a SAFF only in 39 (81.3%) cases, while in the remaining nine (18.8%) patients a SAFF slip was combined with a LD flap. In the majority of patients (*n* = 35; 72.9%), one to three slips were used among the fifth to ninth serratus' slips for facial soft tissue augmentation. One Japanese‐group chose even superficial subslips for dynamic facial reconstruction in the 13 remaining cases.

**FIGURE 2:**
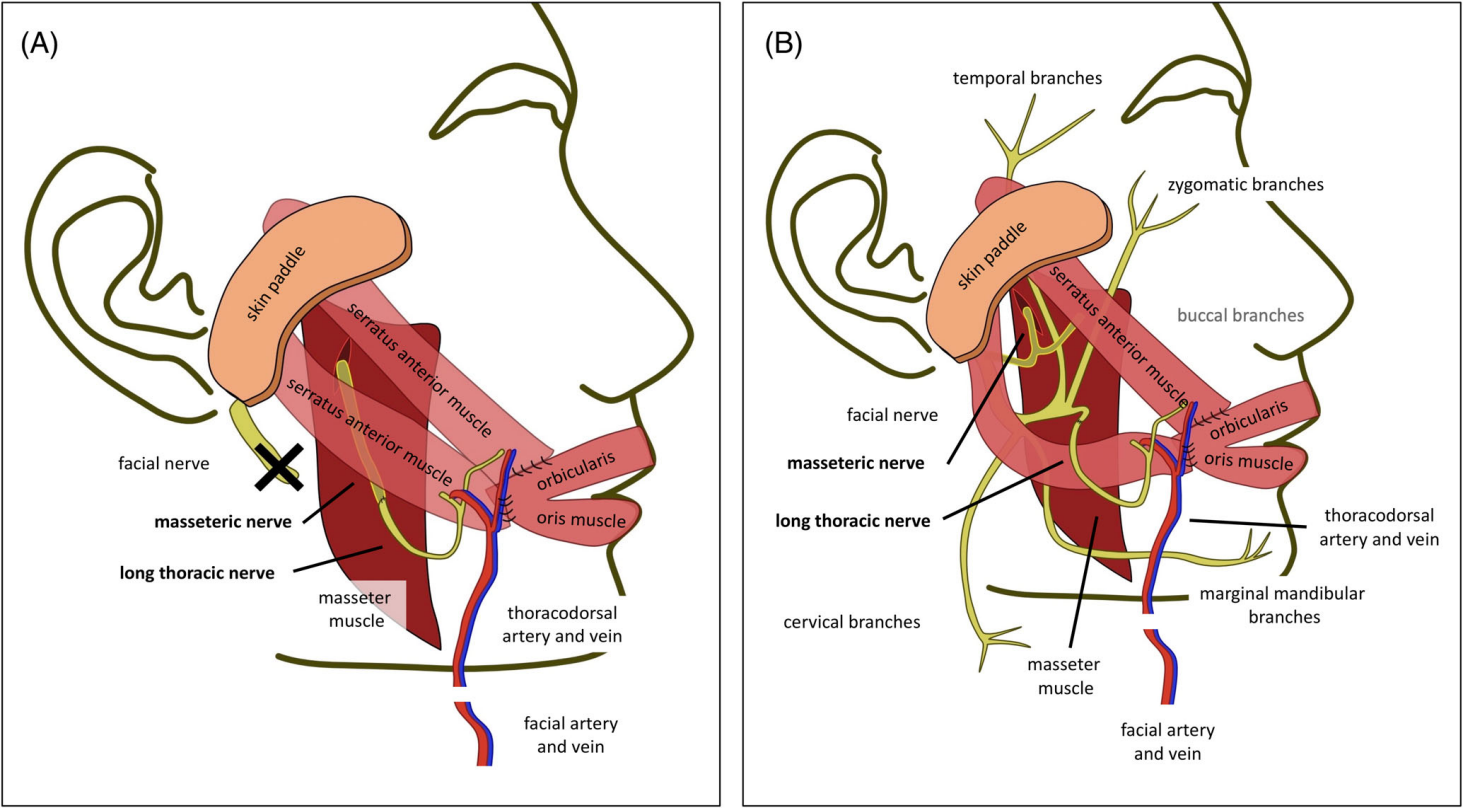
Composite SAFF for immediate facial reanimation. A composite serratus anterior free flap (SAFF) with two slips (two muscle vectors) is used for immediate dynamic facial reanimation after radical parotidectomy with facial nerve resection (A) or resection of the buccal branches (B). The long thoracic nerve (LTN) was anastomosed with the masseteric nerve (MN) alone (A) for a single‐innervation. Conversely, the MN was directly anastomosed to the SA and the LTN was end‐to‐end anastomosed to the resected buccal branch to achieve a dually‐innervated free flap (B) [Color figure can be viewed at wileyonlinelibrary.com]

From a surgical point of view, the distance between the root of the helix and the modiolus at rest on the healthy patient's side was used as reference length for the required muscle slip, which ranged from 6 to 20 cm. If two muscle slips were used, the scapular origin (proximal third) of the slips was fixed to the middle‐third of the upper lip, the modiolus, and the upper third of the lower lip (Figure 2). The opposite sides may further be trimmed if required and sutured to the preauricular or deep temporal fascia to achieve a 40–50° angle of the upper and a 10–20° angle of the lower slip regarding to a horizontal plane. Myocutaneous composite flaps were reported only by one group for four patients (8.3%). Further characteristics of harvested SAFFs are summarized in Table 2.

**TABLE 2:** Characteristics of used serratus anterior free flaps

Included studies	Flap	Slips	Skin paddle	Procedure	CFNG	Nerval innervation	
Sakuma et al.^25^	SA	2*	0/1	Two‐stage	Sural N.	Dual	LTN end‐to‐end with MN; CFNG end‐to‐side with LTN
Watanabe et al.^26^	SA + LD	1	0/7	One‐stage	TDN	Dual	LTN *end‐to‐end with thinner* MN *branch*; *Thicker* MN *branch intramuscular neurotization with* LD; TDN *end‐to‐end with contralateral* FN
Sakuma et al.^24^	SA	2–3*	0/12	One‐stage	No	Single	LTN *end‐to‐end with* MN
Matsumine et al.^27^	SA + LD	1	0/2	One‐stage	TDN	Dual	LTN *end‐to‐end with* MN *and end‐to‐side with* TDN; TDN *end‐to‐end with contralateral* FN
Gundeslioglu et al.^44^	SA	1	0/5	One‐stage	No	Single	LTN *end‐to‐end with* marginal mandibular branch of FN
Cheng et al.^29^	SA	NA	0/4	One‐stage	No	Single	LTN *end‐to‐end with* cervical branch of FN
Tang et al.^45^	SA	NA	4/6	One‐stage	No	Dual	LTN *end‐to‐end with* FN trunk; *intramuscular neurotization of thinner* FN branches *with* SA
Leonetti et al.^21^	SA	2–3	0/3	One‐stage^a^	Sural N.^b^	Single	LTN *end‐to‐end with fascicles of the* facial nerve trunk
Yoleri^28^	SA	3	0/2	Two‐stage	Sural N.	Single	LTN *end‐to‐end with contralateral* FN via CFNG
Ylä‐Kotola et al.^46^	SA	NA	0/6	Two‐stage	Sural N.	Single	LTN *end‐to‐end with contralateral* FN via CFNG

*Note*: One to three slips were used in the majority of cases. Two groups used even subslips (*) for less bulk. Neurotized free flaps were applied in 4 out of 48 patients (8.3%). Number of procedures and type neurotization are further indicated.

Abbreviations: CFNG, cross facial nerve graft; FN, facial nerve; LD, latissimus dorsi muscle; LTN, long thoracic nerve; NA, not available; SA, serratus anterior muscle; TDN, thoracodorsal nerve.

^a^ Two procedures were performed within 1 week during one‐inpatient stay.

^b^ Sural nerve was used as nonvascularized graft for upper facial reanimation.

### 3.4 Neurotization

Single‐ or dual‐innervated muscle transfers were used in 32 (66.7%) and 16 (33.3%) cases, respectively, which were largely performed as one‐stage procedures (*n* = 39; 81.3%). CFNG using the sural nerve (*n* = 9) or the thoracodorsal nerve (TDN; *n* = 9) was applied in 37.5% of patients. The TDN was used in those nine patients in whom reconstruction was performed with a combined SAFF and LD flap. It is worth emphasizing that CFNG using the sural nerve always required a second procedure, while using the TDN required harvest of the LD muscle as a secondary muscle vector as well. The LTN was coaptated with the masseteric nerve (MN) in 22 patients (45.8%) or directly anastomosed with remaining branches of the facial nerve (FN) in 18 patients (37.5%), including fascicles of the main trunk (*n* = 9), the marginal mandibular branch (*n* = 5), or the cervical branch (*n* = 4), respectively (Table 2 and Figure 2).

### 3.5 Outcome

The mean (median) follow‐up time was 29.8 (23.0) months with a range from 6 to 97 months. Basically, there existed no congruent outcome parameters and sufficient information regarding functional outcome was given only by eight groups providing data of 37 patients. Among those, objective scoring systems (House‐Brackman, Terzis and Noah Score, Harii's criteria) were applied in 26 (70.3%) patients. Moreover, an electromyography (EMG) was additionally used for proof of sufficient neural reanimation in a minority of nine (24.3%) patients. Although comparisons were hampered by inconsistent evaluation of outcomes, satisfying facial reanimation with good to excellent results were reported for all patients. A voluntary contraction was mostly reported as first sign of sufficient reanimation and was first noticed after a mean (median) time of 5.44 (5.0) months after dynamic SAFF transfer.

### 3.6 Donor site morbidity

Information regarding donor site morbidity was provided in nine (*n* = 42) studies (Table 3). Scapular winging or impaired shoulder function did not occur in any of those patients. Flap loss due to venous thrombosis was mentioned in one case (2.4%) indicating a 97.6% flap survival rate. Altogether, revision surgeries were performed in four patients (9.5%) either to reduce muscle bulk (*n* = 2), revise flap failure (*n* = 1) or to reduce muscle slips (*n* = 1) aiming to strengthen labial, preauricular, and temporal ends.

**TABLE 3:** Donor site morbidity and revision surgery

Included study	Nr.	Scapular winging	Impaired shoulder function	Flap loss	Revision surgery	Debulking
Sakuma et al.^25^	1	0 (0)	0 (0)	0 (0)	0 (0)	0 (0)
Watanabe et al.^26^	7	0 (0)	0 (0)	0 (0)	0 (0)	0 (0)
Sakuma et al.^24^	12	0 (0)	0 (0)	0 (0)	0 (0)	0 (0)
Matsumine et al.^27^	2	0 (0)	0 (0)	0 (0)	0 (0)	0 (0)
Gundeslioglu et al.^44^	5	0 (0)	0 (0)	1 (20.0)	1 (20.0)	0 (0)
Cheng et al.^29^	4	0 (0)	0 (0)	0 (0)	0 (0)	2 (50.0)
Tang et al.^45^	6	0 (0)	0 (0)	0 (0)	0 (0)	0 (0)
Leonetti et al.^21^	3	0 (0)	0 (0)	0 (0)	0 (0)	0 (0)
Yoleri^28^	2	0 (0)	0 (0)	0 (0)	1 (50.0)	0 (0)
Ylä‐Kotola et al.^46^	6	NA	NA	NA	NA	NA
Total	48	0 (0)	0 (0)	1 (2.4)	2 (4.8)	2 (4.8)

## 4 DISCUSSION

Tumor resections of the midface and parotid area are significantly mutilating and may end up in defects requiring complex soft and bony tissue augmentation as well as facial nerve reconstruction. The SAFF flap, either harvested with skin paddle or different muscle vectors or bone, is predisposed for dynamic facial reanimation with its thin fan shaped fashion. With only 10 studies involving 48 patients and describing dynamic facial reconstruction with a SAFF in various clinical settings, surprisingly little attention has been given to the SAFF in this context to date. Therefore, we conducted this review to evaluate the possibilities, disadvantages and pitfalls of dynamic facial reanimation with a SAFF.

Interestingly, 80% of patients were treated in Japan and China compared to 10% in North America and Europe. This demonstrates that the use of the SAFF for dynamic facial reanimation is more widespread in Asia, but the reasons therefore remain largely elusive. Notably, single‐stage procedures with soft tissue reconstruction and dynamic facial reanimation were performed in 81.3% of cases. This is of particular interest as two‐stage procedures are less suited to immediate facial reanimation after radical parotidectomy in mostly elderly patients, need for adjuvant therapy and limited life expectancy.^7^, ^8^ Voluntary contraction as first sign of sufficient reanimation was noticed after 5–6 months, which is remarkably faster compared to two‐stage GMFT usually performed 9–12 months after CFNG. Notably, postoperative radiotherapy did not affect final facial outcome and represents therefore no contraindication for not attempting immediate dynamic facial reconstruction.^20^, ^21^

Satisfying facial reanimation with good to excellent results was reported by all authors with a free flap survival rate of 97.6%. The data demonstrated both the reliability as well as the flexibility of neurotized composite SAFFs in different clinical settings. However, the absence of standardized outcome measures for functional parameters or objective standardized 3D systems^22^, ^23^ as well as small patient cohorts certainly hamper functional analysis and represents therefore a limitation of the study. Nonetheless, functional and aesthetic outcomes do not seem to significantly differ among single‐ and two‐stage procedures. This led us to believe that both procedures were equally effective in terms of outcome.

Another advantageous feature of the SAFF represents its reliable neurovascular pedicle and anatomy allowing harvest of up to five slips based on one single neurovascular pedicle.^12^, ^17^, ^18^, ^24^ Usually, one to three serratus slips, occasionally subslips, were used for soft tissue augmentation.^21^, ^24^, ^25^, ^26^, ^27^, ^28^ Debulking was only performed in two patients with Parry–Romberg syndrom,^29^ which principally indicates a good ratio of demand and supply. As summarized in Table 4, muscle bulk of SAFF is similar to those of GMFT regarding length, width, and thickness.

**TABLE 4:** Comparison of gracilis muscle free transfer and serratus anterior free flap

Characteristics	GMFT	SAFF
Experience	High^23^, ^31^, ^32^	Low^24^, ^25^, ^26^, ^27^, ^29^, ^45^
Procedure	One or two‐step^23^, ^31^, ^32^	Mainly one‐step^24^, ^25^, ^26^, ^27^, ^29^, ^45^
Muscle vectors	1 muscle	1 to 5 muscle slips^17^, ^18^, ^47^
Length of muscle	8 cm (−14 cm)^32^	10 cm (−17 cm)^17^, ^18^, ^47^
Width	3.6 cm^32^	2.4 cm^17^, ^18^, ^47^
Thickness	0.71 cm^32^	0.73 cm^17^, ^18^, ^47^
Skin paddle	Yes^23^, ^31^, ^32^	Yes^15^, ^29^
Force vector^a^	0.963 pounds^47^	0.178 pounds^47^
Length of donor nerve	11–14.8 cm^48^	4–6 cm^24^, ^25^, ^26^, ^27^, ^29^, ^45^
Neurotization	Nerve‐to‐nerve/muscle^23^, ^31^, ^32^	Nerve‐to‐nerve^28^, ^29^, ^30^, ^31^, ^33^, ^45^
Length of pedicle	6 cm^49^	6.5–12 cm^26^
Diameter of vessels	1–2 mm^49^	2–3 mm^18^
Donor site morbidity	Low^23^, ^31^, ^32^	Low to moderate^15^, ^24^, ^25^, ^26^, ^27^, ^29^, ^45^

Abbreviations: GMFT, gracilis muscle free transfer; SAFF, serratus anterior free flap.

^a^ According to Lifchez et al.^47^ smiling generates a maximum force of 0.307 pounds.

Different muscle slips allow creation of different, separate muscle vectors as illustrated in Figure 2. Mostly, two slips were used to restore the risorius and the zygomaticus major muscle, which functionally belong to midface and lower face.^25^, ^27^ In particular, the scapular origins (proximal third) of the muscle slips are positioned on the oral commissure side and fixed at the orbicularis oris muscle of the upper lip (zygomaticus major muscle) and the modiolus (risorius muscle). The opposite sides may be trimmed and secured to the deep temporal and preauricular fascia to achieve a 40–50° angle of the upper and a 10–20° angle of the lower slip (Figure 2).^25^, ^28^ The versatility of the flap and the possibility to include multiple components (bone, skin, muscle) as chimeric flap based on one neurovascular pedicle represents a big advantage of the SAFF over the GMFT (Table 4).

Donor site morbidity and potential long‐term impairments accompanied by free flap harvest and whether these given opportunities may outweigh possible harms need to be considered as well. The free GFMT has a neglectable low donor site morbidity.^22^, ^23^, ^30^, ^31^, ^32^ Similarly, harvest‐related morbidities are extremely rare after SAFF use and signs of scapular winging are seldom reported,^33^ while the overwhelming majority of patients show no difference regarding upper extremity function compared to healthy controls.^16^, ^34^, ^35^, ^36^, ^37^ Therefore, the SAFF is considered as favorable option regarding donor site morbidity compared to other flaps.^38^

The LTN is typically harvested with a length of 4–6 cm, which smoothly allows coaptation with the ipsilateral MN, remaining branches of the facial nerve or the main trunk itself. The ipsilateral facial nerve should be of course favored over ipsilateral MN or CFNG as it offers the most natural option for microvascular anastomosis.^21^ Yet comparisons between CFNG and ipsilateral MN nerval anastomosis in GFMTs have demonstrated no differences regarding failed procedures and greater excursions after ipsilateral MN anastomosis, but resulted superior in symmetry after CFNG.^30^

Most recent data further indicates that dually innervated muscle transfers may provide even better functional outcome compared to single innervated muscle transfers. However, meta‐analyses failed to demonstrate statistically significant benefits.^31^, ^32^, ^39^ According to current data, we would therefore recommend to primarily perform end‐to‐end anastomosis of the LTN with fascicles or branches of the ipsilateral facial nerve and secondly coadaptation with the ipsilateral MN (Figure 2A,B). Whether patients may further benefit from additional intramuscular neurotization by the MN to achieve dual innervation seems likely but needs to be subject of subsequent studies.

The low number of appropriate studies, the small patient cohort and the heterogeneity of outcome measures hamper serious comparisons and represent the main limitation of our review. However, the comprehensive literature review demonstrating the versatility and reliability of the SAFF for dynamic facial reanimation in different clinical settings represent the strength of our work. Compared to the GMFT, which represents the gold‐standard for dynamic facial reanimation, the SAFF provides similar muscle bulk and a reliable, long neurovascular pedicle. The option for skin paddles^14^, ^15^, ^16^, ^40^, ^41^, ^42^, ^43^ and especially the creation of multiple predictable force vectors represent strong arguments for using the SAFF for immediate dynamic facial reanimation after ablative parotid surgery. Some surgeons even argue against the SAFF because of the need of repositioning after ablative surgery and the supposed significant time delay. By using a balloon under the latissimus dorsi muscle for lateral positioning, a two‐teams approach is possible and the flap harvest can be done appropriately and in a timely manner. We hope that our work will encourage further application and research of the SAFF to steadily evaluate its potential and limitations.

## 5 CONCLUSION

The serratus anterior free flap with its long neurovascular pedicle, the fan‐shaped muscle slips, and reliable skin paddles, represents an excellent candidate for dynamic facial reanimation after ablative midface and parotid surgeries.

## CONFLICT OF INTEREST

The authors declare that there is no conflict of interest that could be perceived as prejudicing the impartiality of the research reported.

## Data Availability

The data that support the findings of this study are available from the corresponding author upon reasonable request.
